# Comparison of Embryo and Egg Quality in Polycystic Ovary Syndrome Patients Having Regular vs Irregular Menstrual Patterns

**DOI:** 10.7759/cureus.99597

**Published:** 2025-12-19

**Authors:** Faza Fatima, Rohina Gul, Shehzadi Imrana, Aisha Awais, Marium Mustanser, Nighat Mahmood, Saba Sardar, Rameen Nisar

**Affiliations:** 1 Obstetrics and Gynaecology, Hameed Latif Hospital, Lahore, PAK; 2 Fertility, Hameed Latif Hospital, Lahore, PAK; 3 In Vitro Fertilization, Hameed Latif Hospital, Lahore, PAK; 4 Clinical Embryology, Hameed Latif Hospital, Lahore, PAK; 5 Assisted Reproductive Technology, Hameed Latif Hospital, Lahore, PAK; 6 Research, Hameed Latif Hospital, Lahore, PAK

**Keywords:** embryo quality, intra cytoplasmic sperm injection, in vitro fertilization, menstrual pattern, oocyte competence, polycystic ovarian syndrome

## Abstract

Introduction: Polycystic ovary syndrome (PCOS) presents with heterogeneous phenotypes that may influence reproductive outcomes in assisted reproductive treatment. To compare oocyte and embryo quality in PCOS patients with regular versus irregular menstrual cycles undergoing in vitro fertilization (IVF)/intracytoplasmic sperm injection (ICSI) using a uniform stimulation protocol.

Material and methods: This retrospective cohort study was conducted at an infertility clinic in Lahore, Pakistan, over 12 months (January-December 2024). PCOS patients aged 25-39 years undergoing their first IVF/ICSI cycle with a short antagonist protocol were included and stratified into regular and irregular cycle groups. Baseline characteristics, stimulation response, oocyte yield, fertilization and cleavage rates, and blastocyst-stage development were compared.

Results: A total of 50 patients met the inclusion criteria (19 regular cycle, 31 irregular cycle). Irregular cycle patients required higher gonadotropin doses and exhibited greater hormonal dysregulation. Although oocyte maturation was higher in the irregular group, blastocyst-stage embryo development was more frequent in the regular cycle group, indicating superior downstream competence. Fertilization and cleavage outcomes were comparable between groups.

Conclusion: Menstrual irregularity in PCOS is associated with a less favorable embryo development profile despite similar initial fertilization kinetics. PCOS patients with regular menstrual cycles exhibited a higher yield of advanced-stage embryos, suggesting a milder reproductive phenotype.

## Introduction

PCOS constitutes the most prevalent endocrine disorder that is practiced in gynecology, especially reproductive endocrinology and subfertility clinics. It has been reported to be alarmingly high in Pakistan, cited at 50-55.4% [1]. The amended Rotterdam consensus has PCOS with evidence-based criteria of at least two of the following needs: (a) clinical or biochemical hyperandrogenism, (b) ovulatory dysfunction, and (c) polycystic ovary morphology (PCOM) or high anti-Mullerian hormone (AMH) [2]. Remarkably, in the case of menstrual irregularity and hyperandrogenism, ultrasound or AMH confirmation is not obligatory [3]. On the other hand, there are women with normal cycles who are not diagnosed until PCOM is identified together with anovulation, either by no growth of follicles on sonogram or low levels of progesterone in serum samples. Although this is a common diagnostic umbrella, PCOS is a very heterogeneous disorder [4]. Regularity of the cycles of patients classifies them into specific phenotypes, which can vary in terms of endocrine milieu, insulin resistance, follicular kinetics, and reproductive competence [5]. More pronounced maladjustments in metabolism and androgenicity are also likely to be found in women with irregular cycles, which may hamper oocyte maturation and embryo competence. Conversely, comparatively intact ovarian ability and, possibly, gametal and embryonic quality, could be maintained by normal menstruating PCOS women, who often represent a milder phenotype [6].

In addition to having distinct menstrual cycles, the ovulatory and anovulatory PCOS phenotypes differ in hormonal, metabolic, and oocyte-level characteristics. Females who have irregular menstrual cycles are more often characterized by marked hyperandrogenism, insulin resistance, and abnormal secretion of gonadotropin [7]. These systemic abnormalities alter the intrafollicular environment, hindering oocyte maturation, spindle formation, and mitochondrial competence, even when mature oocytes are retrieved [8]. Conversely, it is possible that PCOS patients with normal cycles have milder endocrine disruption and could be better oocyte competent and embryo growing despite the same ovarian morphology [9]. The quality of embryos in assisted reproductive technology (ART) is a direct by-product of oocyte quality. A compromised follicular fluid composition, oxidative stress burden, and excess androgen may affect the potential to fertilize, cleavage kinetics, and blastocyst formation. As a result, the embryo grade, the embryo of usability, and implantation probability can vary greatly in PCOS women who have normal versus abnormal cycles [10].

PCOS patients have been managed as a homogeneous group in the ART practice. All stimulation protocols, trigger strategies, and expectations of embryo yield are generally used without stratification according to menstrual regularity [11]. When intrinsic oocyte and embryo quality are connected with menstrual pattern, it is possible that by disregarding this phenotype difference, suboptimal counseling, incompatible expectations, and non-individualized ovarian stimulation regimens are obtained. The question of whether or not menstrual regularity is a diagnostic finding or a biological predictor of gametogenic competence is, therefore, a significant clinical issue [12]. Exaggerated follicular response and the risk of developing ovarian hyperstimulation syndrome are the common features of controlled ovarian hyperstimulation in PCOS patients with irregular cycles [13]. Individuals may have a more moderate response and possibly improved downstream embryo viability. An egg and embryo quality head-to-head comparison of the two phenotypes can help to understand whether menstrual regularity is a useful surrogate measure when making ART decisions [14]. Although clinically relevant, there is little research on a direct comparison of oocyte maturity, fertilization rate, and embryo grade in PCOS patients stratified by menstrual cyclicity [15]. The majority of the existing literature has lumped all the phenotypes of PCOS, concealing the possible variation in the intrinsic reproductive competence. This gap can be filled by a phenotype-based comparison where the researcher finds out whether the evolution of regular cycles is predictive of better embryo potential regardless of ovarian morphological characteristics [16].

Assisted reproduction is often the final therapeutic resort for PCOS-associated infertility in Pakistan. In vitro fertilization (IVF)/intracytoplasmic sperm injection (ICSI) is effective in this population, yet patients commonly self-fund treatment without insurance coverage, often after exhausting both unregulated and formal medical options. Given the financial, psychological, and temporal cost of ART in Pakistan, phenotype-specific prognostic counseling is crucial. Because PCOS is highly prevalent among subfertile women undergoing IVF/ICSI locally, understanding differential egg and embryo quality between those with regular versus irregular menstrual cycles is clinically significant. Such evidence could refine expectations, guide individualized ovarian stimulation, and inform counseling in Pakistani cohorts where cultural, dietary, genetic, and healthcare access factors may modify outcomes. This study aimed to compare the embryo and egg quality in polycystic ovary syndrome patients having regular vs irregular menstrual patterns.

## Materials and methods

This was a retrospective cohort study conducted at Lahore Institute of Fertility and Endocrinology (LIFE) Infertility Clinic, Hameed Latif Hospital, Lahore, Pakistan, from January 2024 to December 2024. All patients included in this study underwent IVF or ICSI cycles at the same center. The study was approved by the Research Ethics Committee of Lahore Institute of Fertility and Endocrinology of the institute (reference number: IRB/LIFE/2023/22).

Study population and sample size

All the patients who were diagnosed with PCOS based on at least two of the three Rotterdam criteria, age between 25 and 39 years, Regular menstrual cycles (21-35 days) or irregular cycles (>35 days or non-patterned), body mass index below 40 kg/m², undergoing first ICSI cycle and ovarian stimulation with the short stimulation protocol (start ovarian stimulation with gonadotropin injections on days 2-3 of the cycle) were included. Patients with concurrent infertility factors, including endometriosis, male factor infertility, tubal factor infertility, adenomyosis, pelvic tuberculosis, or prior ovarian surgery, lost to follow-up and did not proceed to ovum pickup were excluded. 

The sample size was 50, which was calculated by the G-POWER software with the effect size of 0.50 and power of 0.95. 

Data collection

Informed consent was obtained before the data collection. Patients meeting eligibility criteria were classified into two groups according to menstrual pattern: regular cycle group and delayed or irregular cycle group. Short protocol stimulation was initiated on day 2 or 3 of the menstrual cycle. Baseline reproductive hormones (follicle-stimulating hormone (FSH) (IU/L), luteinizing hormone (LH) (IU/L), prolactin (ng/mL), and thyroid-stimulating hormone (TSH) (mIU/L)) were assessed before stimulation. Patients received either recombinant follitropin or human menopausal gonadotropin, alone or in combination. Follicular monitoring was performed using transvaginal ultrasonography. A gonadotropin-releasing hormone (GnRH) antagonist was added once the lead follicles reached 12-14 mm. When the major follicular cohort reached 16-22 mm, ovulation was triggered using either human chorionic gonadotropin (5000 IU or 10,000 IU) or a GnRH agonist trigger (decapeptyl 0.2 mg). Oocyte retrieval was carried out 35-36 hours post trigger.

Fertilization, embryo culture, and outcome measures

Retrieved oocytes were classified as mature (M2) or immature (M1) according to standard embryology criteria. ICSI was performed on M2 oocytes. Fertilization was assessed the following day, and cleavage-stage development was recorded. Embryo grading was performed on day 5, and good-quality embryos were cryopreserved according to institutional protocol. Ratios of follicles to oocytes, fertilization rate, cleavage rate, and final embryo grade were recorded as outcome variables.

Statistical analysis

All data were analyzed using IBM SPSS Statistics for Windows, version 25 (IBM Corp., Armonk, New York, United States). Continuous variables, normally distributed, were expressed as mean and standard deviation. Student t-test was used to compare two groups according to the distribution of the variable. A p-value ≤ 0.05 was considered statistically significant. Multivariate logistic regression was applied for multiple independent variables to check the relationship with the dependent variable. 

Multicollinearity was evaluated using the variance inflation factor (VIF). All predictors demonstrated acceptable VIF values (< 5), indicating the absence of problematic multicollinearity. Therefore, all variables were retained in the multivariable logistic regression model.

## Results

**Figure 1:**
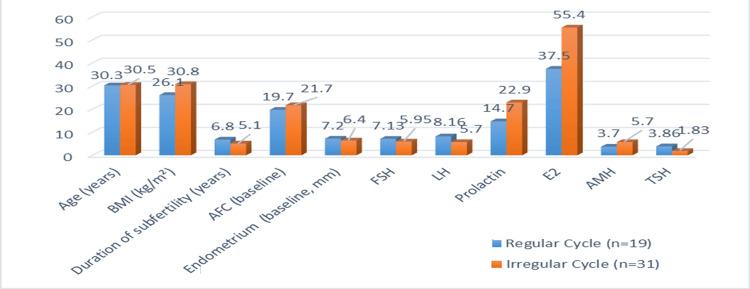
Comparison of baseline characteristics of patients Data presented as mean values BMI: body mass index; FSH: follicle-stimulating hormone; LH: luteinizing hormone; AMH: anti-Mullerian hormone; TSH: thyroid-stimulating hormone; AFC: antral follicle count

The mean age of the women in both groups was 30 years. The regular cycle group had a slightly lower BMI (26.1 ± 3.79) than the irregular group (30.8 ± 25.63). The menstrual cycle exhibited a significant difference with the BMI, although it was not statistically significant with age (p-value=0.00, 0.88), respectively. The duration of subfertility was almost similar between groups (6.8 ± 3.23 vs. 5.1 ± 2.7 years, p=0.05). Antral follicle count (AFC) showed a meaningful difference (19.7 ± 6.36 vs. 21.7 ± 2.59). The baseline endometrial thickness was markedly higher in the regular group (7.24 ± 4.18 mm) versus the irregular group (6.4 ± 1.61 mm). The regular group also had higher LH (8.16 ± 6.79 vs. 5.7 ± 3.98) and TSH (3.86 ± 6.54 vs. 1.83 ± 0.96), and significantly lower AMH (3.7 ± 2.15 vs. 5.70 ± 4.03, p=0.06) (Table 1, Figure 1).

**Table 1:** Comparision of baseline characteristics of participants with regular menstrual cycles and irregular menstrual cycles Student t-test was used to compare two groups according to the distribution of the variable. The significance level was p < 0.05. BMI: body mass index; FSH: follicle-stimulating hormone; LH: luteinizing hormone (LH); AMH: anti-Mullerian hormone; TSH: thyroid-stimulating hormone; AFC: antral follicle count

Parameter	Regular Cycle (n=19), mean ± SD	Irregular Cycle (n=31), mean ± SD	t-value	p-value
Age (years)	30.3 ± 4.30	30.5 ± 3.99	-0.14	0.88
BMI (kg/m²)	26.1 ± 3.79	30.8 ± 5.63	-3.23	0.00*
Duration of subfertility (years)	6.8 ± 3.23	5.1 ± 2.77	1.98	0.05
AFC at first scan	19.7 ± 6.36	21.7 ± 2.59	-1.56	0.12
Endometrial thickness (mm)	7.2 ± 4.18	6.4 ± 1.61	0.95	0.33
FSH (IU/L)	7.13 ± 3.17	5.95 ± 5.17	0.89	0.37
LH (IU/L)	8.16 ± 6.79	5.7 ± 3.98	-0.16	0.11
Prolactin (ng/mL)	14.7 ± 6.57	22.9 ± 30.10	-1.03	0.31
Estradiol (pg/mL)	37.5 ± 5.58	55.4 ± 26.7	-0.89	0.39
AMH (ng/mL)	3.7 ± 2.15	5.70 ± 4.03	-1.91	0.06
TSH (mIU/L)	3.86 ± 6.54	1.83 ± 0.96	1.65	0.10

Patients with regular menstrual cycles required a lower total gonadotropin dose (1926.6 ± 669.1 IU) compared with the group with irregular cycles (2398.6 ± 1379.1 IU). Stimulation duration was comparable (13.7 ± 1.36 vs. 14.3 ± 1.74 days). Endometrial thickness on trigger day was almost similar in both groups. Estradiol on the trigger day was higher in the irregular group as compared to the regular group (3652.6 ± 931.9 vs. 3360.1 ± 1127.1). The progesterone level was 5.30 ± 1.73 vs. 5.44 ± 2.12, reflecting a stronger hormonal response to stimulation (Table 2).

**Table 2:** Comparision of stimulation characteristics of the two groups Student t-test was used to compare two groups according to distribution of the variable. The significance level was p < 0.05. GnRH: gonadotropin-releasing hormone

Parameter	Regular Cycle (n=19), mean ± SD	Irregular Cycle (n=31), mean ± SD	t value	p-value
Total GnRH dose (IU)	1926.6 ± 669.1	2398.6 ± 1379.1	1.62	0.11
Days of stimulation	13.7 ± 1.36	14.3 ± 1.74	-1.31	0.19
Endometrium on decision day (mm)	9.89 ± 2.05	9.67 ± 1.95	0.37	0.71
Estradiol (pg/mL)	3360.1 ± 1127.1	3652.6 ± 931.9	-0.99	0.32
P4 (nmol/L)	5.3 ± 1.73	5.44 ± 2.12	-0.10	0.92

Although the number of follicles was comparable (21.4 ± 6.03 vs. 23.6 ± 4.90), fewer oocytes were retrieved in the regular group (12.1 ± 5.19) compared with the irregular group (15.5 ± 7.80). Mature oocytes were slightly more in the irregular group (10.8± 5.27 vs. 12.1 ± 7.32), but this was not significant. Fertilized oocytes (8.73 ± 4.44 vs. 8.75 ± 5.88, p=0.98) and cleavage stage embryos (8.5± 4.64 vs. 8.10 ± 5.65, p=0.78) were not statistically different, indicating similar early embryonic kinetics in both groups (Table 3).

**Table 3:** Follicular and embryology outcomes The significance level was p < 0.05. The data was presented in the form of mean and standard deviation. Student t-test was used to compare two groups according to distribution of the variable.

Parameter	Regular Cycle (n=19), mean ± SD	Irregular Cycle (n=31), mean ± SD	t-value	p-value
Number of follicles	21.4 ± 6.03	23.6 ± 4.90	-1.37	0.17
Oocytes retrieved	12.1 ± 5.19	15.5 ± 7.80	-1.69	0.09
Mature oocytes	10.8 ± 5.27	12.1 ± 7.32	-1.15	0.25
Fertilized oocytes	8.7 ± 4.44	8.8 ± 5.88	-0.01	0.98
Cleaved embryos	8.5 ± 4.64	8.10 ± 5.65	0.27	0.78

**Figure 2:**
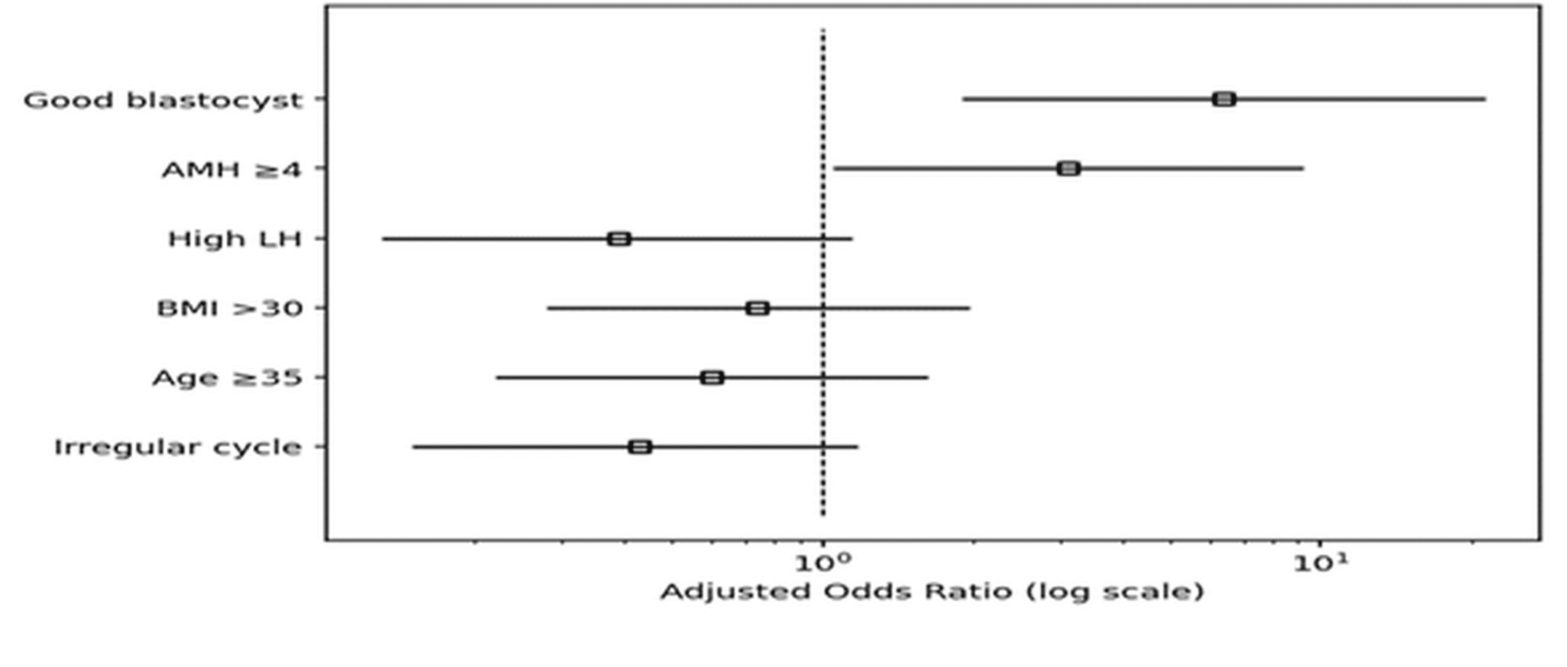
Forest plot showing strongest positive predictors of clinical pregnancy The strongest positive predictors of clinical pregnancy were the availability of a good-quality blastocyst and AMH ≥4 ng/mL, both with odds ratios clearly above 1. Factors such as irregular cycles, high LH, high BMI, and age ≥35 lowered the odds of pregnancy, but their confidence intervals crossed 1, indicating they were not statistically significant predictors in this model. BMI: body mass index; AMH: anti-Mullerian hormone; LH: luteinizing hormone

Good-quality blastocyst availability (adjusted OR (aOR) 6.40, 95%CI 1.90-21.4, p=0.002) and AMH ≥4 ng/mL (aOR 3.12, 95%CI 1.05-9.22, p=0.04) independently increased the odds of clinical pregnancy. On the other hand, although they did not achieve significance, irregular cycles, high LH, age ≥35, and BMI>30 all decreased pregnancy risks (aOR 0.43). VIF values indicated moderate multicollinearity (Table 4, Figure 2).

**Table 4:** Multivariable logistic regression analysis for clinical pregnancy BMI: body mass index; LH: luteinizing hormone; AMH: anti-Mullerian hormone

Predictor Variable	β Coefficient	Adjusted Odds Ratio (AOR)	95% CI	p-value
Irregular menstrual cycle (ref = regular)	−0.84	0.43	0.15 – 1.18	0.09
Age ≥ 35 years	−0.51	0.60	0.22 – 1.63	0.32
BMI > 30 kg/m²	−0.29	0.74	0.28 – 1.97	0.54
High LH (>6 IU/L)	−0.93	0.39	0.13 – 1.14	0.08
AMH ≥ 4 ng/mL	+1.14	3.12	1.05 – 9.22	0.04*
Good-quality blastocyst available	+1.86	6.40	1.90 – 21.4	0.002*

## Discussion

This study involved a comparison of the quality of embryos and oocytes of patients with PCOS who underwent IVF/ICSI according to their menstrual patterns. Despite having the same etiologic diagnosis of PCOS, the data demonstrated notable clinical differences between women with regular and irregular cycles. Patients with irregular menstrual cycles had higher gonadotropin requirements and more evident hormonal imbalances. This corresponds to a more metabolically and endocrinologically extreme phenotype, which could be the cause of increased stimulation doses and changed endometrial reaction. Even though the total amount of follicles did not differ between groups, patients with irregular cycles exhibited fewer retrieved oocytes, indicating that follicular recruitment does not necessarily result in the same harvest in this subgroup. Interestingly, the irregular group had a higher oocyte maturation rate, although the fertilization and cleavage rates were similar among groups, despite having a smaller number of oocytes. These results indicate that preimplantation ability is not necessarily linked to menstrual cyclicity, and irregularity does not necessarily disrupt early fertilization processes [17].

According to the guidelines of the National Institute for Health and Care Excellence (NICE), patients with PCOs diagnosed and already receiving up to 12 cycles of ovulation induction can receive laparoscopic ovarian drilling or proceed to IVF/ICSI [18]. In Pakistan, most of the couples who are experiencing subfertility have already tried various treatments from various medical practitioners and even unqualified quacks and charlatans before they can get to know about the ART methods, such as IVF/ICSI. Therefore, patients receiving this type of treatment should be informed about their opportunities and complications in a comprehensive form and provided with the necessary recommendations, such as lifestyle change and weight loss in case of necessity [19]. Many times, patients are unaware that they have PCOS, as their cycles are regular; however, they may be having an ovulatory cycle as depicted by low luteal phase progesterone levels of follicular tracking on ultrasound, or they may have features of PCOS on ultrasound only, along with symptoms of hyperandrogenism. As two out of three Rotterdam criteria are considered sufficient to make the diagnosis, planning the treatment becomes easier [20].

This study was aimed at finding out the differences in egg and embryo quality in two groups of patients. The aim was to provide more clarity for both the clinician as well as the patients on what to expect in these two different groups. A patient labelled as having PCOS was then assigned to either the group having regular cycles lasting between 21 and 35 days. The patients with irregular cycles had a cycle of more than 35 days or had to have withdrawal bleeds and had no specific pattern of cycles [21]. Patients underwent their routine workup before the stimulation cycle, which ruled out any other coexisting problems such as thyroid disorders and male factor. The stimulation cycle was started as a short protocol with regular follicular monitoring. Proper counselling regarding ovarian hyperstimulation was done, considering that PCOS itself is a risk factor for this condition. GnRH agonist protocol /long protocol was avoided as per the latest European Society of Human Reproduction and Embryology (ESHRE) guidelines [22-24,25]. Patients with irregular cycles had to be given a longer cycle of injections, a greater dosage overall, and hence had higher E2 levels on the day of trigger as well as endometrial thickness. The patient's egg collection was done at 35 hours of hcg trigger once optimal sizes were achieved of the main follicle cohort, 16-22mm [26]. This possibly shows the molecular level of folliculogenesis better in those having a regular cycle as compared to delayed cycles. A greater number of mature oocytes were seen in group B. Maturity of the eggs is also related to the number of days of stimulation and higher dosage. The overall embryo development and quality were also seen more in the patients having regular cycles, again reinforcing better potential on the side of the egg [27].

This study has several limitations that must be considered when interpreting the findings. First, its retrospective design limits the ability to establish causality and is inherently prone to selection bias and incomplete data recording. Second, the sample size was modest, particularly after stratification of patients by menstrual pattern, which may have reduced statistical power for some comparisons and increased the risk of type II error. Important confounders such as insulin resistance, androgen profile severity, lifestyle factors, and medication exposure before ART were not controlled for, even though these variables may influence oocyte competence and endometrial receptivity. Finally, all data were derived from a single infertility center, which may limit external validity and reduce the applicability of findings to other populations, protocols, or laboratory conditions.

## Conclusions

Menstrual patterns in PCOS patients having IVF/ICSI may indicate biologically significant variations in reproductive capacity, it is determined. Despite similar fertilisation and cleavage results, women with irregular cycles showed more hormonal dysregulation and needed larger doses of gonadotropin, but they also generated fewer blastocyst-stage embryos. On the other hand, a greater percentage of advanced-stage embryos were produced by PCOS patients having regular cycles, indicating better downstream developmental competence.

Cycle regularity itself shows better outcomes in IVF/ICSI, and hence, patients with irregular cycles should be offered strategies such as lifestyle modification and weight reduction, which result in better regularity, thereby improving their results in fertility treatments such as IVF/ICSI. Patients should be educated about their condition so that they not only get the best chance of conceiving but also improve their overall health.
